# Comparative analysis of pathogen distribution in patients with fracture-related infection and periprosthetic joint infection: a retrospective study

**DOI:** 10.1186/s12891-023-06210-6

**Published:** 2023-02-13

**Authors:** Tiancong Ma, Jinyang Lyu, Jingchun Ma, Xin Huang, Kangming Chen, Siqun Wang, Yibing Wei, Jingsheng Shi, Jun Xia, Guanglei Zhao, Gangyong Huang

**Affiliations:** 1 Department of Orthopaedic Surgery Huashan Hospital Fudan University, 12Th Wulumuqi Middle Road, Jing’an District, Shanghai, China; 2 Fudan University, 220Th Handan Road, Yang’pu District, Shanghai, China; 3 Department of Orthopaedic Surgery North Branch of Huashan Hospital Fudan University, 518Th Jingpohu Road, Bao’shan District, Shanghai, China

**Keywords:** Pathogen distribution, Periprosthetic joint infection, Fracture-related infection, Microbial pattern, Empirical antimicrobial therapy

## Abstract

**Background:**

The purpose of this study is to investigate the microbial patterns of periprosthetic joint infection (PJI) and fracture-related infection (FRI), and guide for the formulation of more accurate empirical antimicrobial regimens based on the differences in pathogen distribution.

**Methods:**

A comparative analysis of pathogen distribution was conducted between 153 patients (76 with PJI and 77 with FRI). Predicted analyses against isolated pathogens from two cohorts were conducted to evaluate the best expected efficacy of empirical antimicrobial regimens (﻿imipenem + vancomycin, ciprofloxacin + vancomycin, and piperacillin/tazobactam + vancomycin).

**Results:**

Our study found significant differences in pathogen distribution between the PJI and FRI cohorts. *Staphylococci* (61.3% vs. 31.9%, *p* = 0.001) and Gram-negative bacilli (GNB, 26.7% vs. 56.4%, *p* < 0.001) were responsible for the majority of infections both in the PJI and FRI cohorts, and their distribution in the two cohorts showed a significant difference (*p* < 0.001). Multi-drug resistant organisms (MDRO) were more frequently detected in the FRI cohort (29.3% vs. 44.7%, *p* = 0.041), while methicillin-resistant coagulase-negative *Staphylococci* (MRCoNS, 26.7% vs. 8.5%, *p* = 0.002) and *Canidia albicans* (8.0% vs. 1.1%, *p* = 0.045) were more frequently detected in the PJI cohort. *Enterobacter spp.* and *Acinetobacter baumannii* were detected only in the FRI cohort (11.7% and 8.5%, respectively).

**Conclusions:**

*Staphylococci* and GNB were responsible for the majority of infections in both PJI and FRI. Empirical antimicrobial therapy should focus on the coverage of *Staphylococci* in PJI and GNB in FRI, and infections caused by MDROs should be more vigilant in FRI, while the high incidence of MRCoNS in PJI should be noted, which could guide for the formulation of more accurate empirical antimicrobial regimens. Targeted therapy for FRI caused by *A. baumannii* and PJI caused by *C. albicans* needs to be further investigated. Our study reports significant differences in pathogen distribution between the two infections and provides clinical evidence for studies on the mechanism of implant-associated infection.

**Supplementary Information:**

The online version contains supplementary material available at 10.1186/s12891-023-06210-6.

## Introduction

Periprosthetic joint infection (PJI) and fracture-related infection (FRI) are both known as implant-associated infections and are devastating complications in arthroplasty and trauma surgery [1]. Unfortunately, patients with PJI or FRI typically require repeated surgical procedures, long-term antimicrobial therapy, and prolonged bone healing time, which may result in poor functional outcomes [2, 3]. PJI is currently the leading complication after arthroplasty, and the overall incidence remains 0.3–1.7% after THA and 0.8–1.9% after TKA [4]. FRI rates after internal fixation range from 1–2% in the case of closed fractures and up to 25–30% in the case of severe open injuries [5, 6]. As the number of arthroplasties and trauma surgeries increases year by year, prevention and treatment of implant-related infections are becoming increasingly important [7, 8].

To date, empirical antimicrobial therapy (EAT) for PJI with glycopeptide in combination with broad-spectrum β-lactams are currently recommended to cover common pathogens such as Gram-positive cocci and Gram-negative bacilli (GNB) [9–11]. Specific recommendations concerning the prevention and treatment of FRI have been proposed [12–14], since a consensus definition for FRI published in 2018 [15]. The differences in microbial epidemiology between PJI and FRI have only been reported once in Germany [12], and it was a single center study, which may lead to a local epidemiological bias. Due to different national, geographical and economic conditions, this single study cannot fully illustrate the differences in pathogen distribution between PJI and FRI in Asia. As a result, the conclusion of empirical antimicrobial regimens needs to be confirmed further to ensure reliability. The purpose of this study was mainly to investigate the microbial patterns of PJI and FRI, determine whether pathogen distribution in PJI and FRI shows differences in our institution, and provide guidance for the formulation of more accurate empirical antimicrobial regimens.

## Materials and methods

### Study design and patient identification

The study was approved by the Ethics Committee of Huashan Hospital, Fudan University (KY2022-803). Informed consent was obtained from all individual participants included in the study. A retrospective review of 153 patients (18 years old and above) treated for FRI or PJI within the period from 1 January 2016 to 28 February 2021 and performed at a single center was conducted. PJI was diagnosed according to the EBJIS definition of PJI, which was supported by MSIS and ESGIAI in 2021 [16]. FRI was diagnosed according to the latest consensus on the definition of FRI in 2018 [15]. Comparative analyses of pathogen distribution were performed between patients with FRI or PJI. Predicted analyses against isolated pathogens from PJI and FRI cohorts were conducted to evaluate the best-expected efficacy of empirical antimicrobial regimens.

### Data collection

By reviewing case histories, information on patients with PJI and FRI was recorded, including sex, age, body mass index (BMI), comorbidities and infection sites. Comorbidities were evaluated by the Charlson Comorbidity Index (CCI) [17]. By reviewing microbiological reports, the results of pathogen detection and antimicrobial susceptibility testing were recorded. In addition, patients with culture-negative infections were included if the patient’s clinical symptoms and examination raised suspicion of PJI or FRI. Each pathogen was documented separately in cases of polymicrobial infection. Multi-drug resistant (MDR) was defined as acquired non-susceptibility to at least one agent in three or more antimicrobial categories [18].

### Microbiological examination

Taken in at least four suspected tissues, two or more intraoperative deep tissue cultures or a combination of preoperative aspiration and intraoperative deep tissue cultures that yield the same organism may be considered definitive evidence of infection. Exceptionally, the growth of a virulent pathogen (e.g., *S. aureus*, *Escherichia coli* and *Pseudomonas aeruginosa*) in a single sample may also represent infection. If a single sample from multiple tissue cultures yields a pathogen considered a common contaminant (e.g., coagulase-negative *Staphylococci*, *Propionibacterium acnes*, and *Corynebacterium spp.*), we did not consider it as evidence of definitive infection, in which case it was evaluated in conjunction with other clinical evidence. Patients were excluded if less than four samples were taken.

### Statistical analysis

Statistical analysis of the results was performed with STATA/SE 16.0 software (USA). Measurement data were statistically described as the mean, standard deviation, and 95% confidence interval (CI); counting data were described as the frequency. Normally distributed variables were analysed using the t-test. Categorical data are presented as proportions, which were analysed with the chi-square test or Fisher’s exact test. All statistical tests were bilateral, and a *p*-value below 0.05 was considered significant.

## Results

### Demographics

The PJI cohort comprised 76 patients, among whom 36 (47.4%) were male and 40 (52.6%) were female (Table 1). The mean age was 68.0 years (65.6–70.6), and the mean BMI was 23.8 ± 3.9 kg/m^2^. Most patients had comorbidities with a median CCI of 1 (range 0–4). PJI mainly occurred at the knee (28, 36.8%) and the hip (48, 63.2%). The mean delay from prosthesis implantation to the onset of infection was 28 days (range: 14 days-16 months). Seventy-seven patients were diagnosed with FRI in total. Overall, 45 (57.7%) of the patients were male and 32 (42.3%) were female. The mean age was 64.7 years (62.1–67.4), the mean BMI was 24.1 ± 3.7 kg/m^2^ and the median CCI was 1 (range 0–3). FRI mainly occurred at the tibia and/or fibula (33, 41.6%), femur (15, 19.5%), or ulna and/or radius (12, 15.6%). The mean delay from initial fracture treatment to onset of infection symptoms was 7 days (range: 3 days-19 days). In comparison, the groups did not differ significantly in sex, age, BMI, or CCI.

**Table 1:** Baseline characteristics of the PJI and FRI cohorts

Characteristic	PJI (*n* = 76)	FRI (*n* = 77)	*p-*value
Demographic data			
Gender (male), n (%)	36 (47.4%)	45 (57.7%)	n.s
Age, years (95% CI)	68.0 (65.6–70.6)	64.7 (62.1–67.4)	n.s
BMI (kg/m^2^)	23.8 ± 3.9	24.1 ± 3.7	n.s
CCI (95% CI)	1 (0–4)	1 (0–3)	n.s
Delay from prosthesis implantation/ trauma to infection, days (95% CI)	28 days (14 days-16 months)	7 days (3 days-19 days)	0.000
Sites, n (%)			
Hip	48 (63.2%)	3 (3.9%)	
Knee	28 (36.8%)	8 (10.4%)	
Clavicle		5 (6.5%)	
Humerus		9 (11.7%)	
Ulna and/or radius		12 (15.6%)	
Femur		15 (19.5%)	
Tibia and/or fibula		33 (41.6%)	
Pedes		11 (14.3%)	
Microbiologic documentation			
Multi-drug resistant, n (%)	22 (29.3%)	42 (44.7%)	0.041
Negative culture, n (%)	26 (34.2%)	18 (23.4%)	n.s
Polymicrobial infection, n (%)	16 (21.1%)	22 (28.6%)	n.s

### Microbiological analysis

**Fig. 1:**
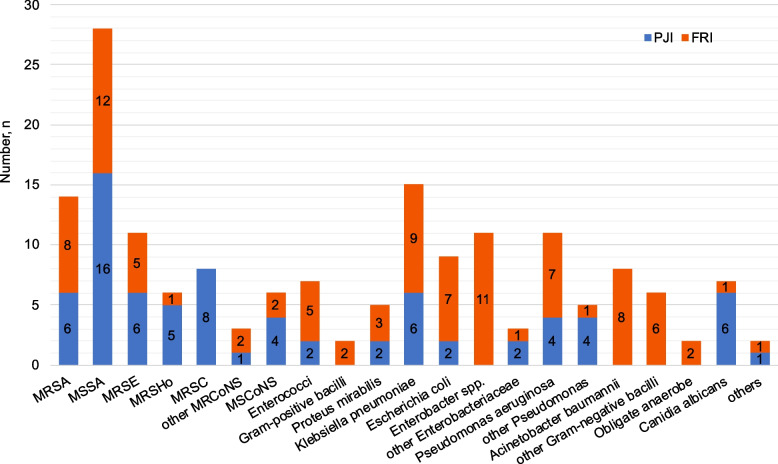
Comparison of isolated micro-organism amounts between PJI and FRI cohorts

MDR organisms (MDRO) were detected in our study, with 22 cases in the PJI cohort and 42 cases in the FRI cohort, resulting in a significant difference (29.3% vs. 44.7%, *p* = 0.041) (Table 1). Both the rate of negative cultures and polymicrobial infections showed no significant difference. Isolated microorganisms of both cohorts are presented in Table 2 and Fig. 1. We also show the isolated microorganisms of the early, delayed and late PJI and FRI respectively (Supplemental Tables 1 and 2).

**Table 2:** Isolated microorganisms in positive culture

Pathogens	PJI (*n* = 75)		FRI (*n* = 94)		*p-*value
Gram-positive cocci	48 (64.0%)		36 (38.3%)		0.001
1 *Staphylococci*	46 (61.3%)		30 (31.9%)		0.000
1.1 *Staphylococcus aureus*	22 (29.3%)		20 (21.3%)		n.s
1.1.1 MRSA	6 (8.0%)		8 (8.5%)		n.s
1.1.2 MSSA	16 (21.3%)		12 (12.8%)		n.s
1.2 CoNS	24 (32.0%)		10 (10.6%)		0.001
1.2.1 MRCoNS	20 (26.7%)		8 (8.5%)		0.002
	6 (8.0%)	MRSE	5 (5.3%)	MRSE	n.s
	5 (6.7%)	MRSHo	1 (1.1%)	MRSHo	n.s
	8 (10.7%)	MRSC			0.001
	1 (1.3%)	*Staphylococcus warneri*			n.s
			2 (2.1%)	MRSH	n.s
1.2.2 MSCoNS	4 (5.3%)		2 (2.1%)		n.s
	4 (5.3%)	MSSE	1 (1.1%)	MSSE	n.s
			1 (1.1%)	MSSHo	n.s
2 *Streptococcus dysgalactiae*	0		1 (1.1%)		n.s
3 *Enterococcus spp.*	2 (2.7%)		5 (5.3%)		n.s
	2 (2.7%)	*Enterococcus faecalis*	3 (3.2%)	*Enterococcus faecalis*	n.s
			2 (2.1%)	*Enterococcus faecium*	n.s
Gram-positive bacilli	0		2 (2.1%)		n.s
1 *Corynebacterium*	0		1 (1.1%)		n.s
2 *Bacillus cereus*	0		1 (1.1%)		n.s
Gram-negative bacilli	20 (26.7%)		53 (56.4%)		0.000
1 *Enterobacteriaceae*	12 (16.0%)		31 (33.0%)		0.012
	2 (2.7%)	*Proteus mirabilis*	3 (3.2%)	*Proteus mirabilis*	n.s
	6 (8.0%)	*Klebsiella pneumoniae*	9 (9.6%)	*Klebsiella pneumoniae*	n.s
	2 (2.7%)	*Escherichia coli*	7 (7.4%)	*Escherichia coli*	n.s
	2 (2.7%)	*Salmonella enteritidis*			n.s
			1 (1.1%)	*Klebsiella oxytoca*	n.s
	0	*Enterobacter*	11 (11.7%)	*Enterobacter spp.*	0.001
			9 (9.6%)	*Enterobacter cloacae*	0.005
			2 (2.1%)	*Enterobacter kobei*	n.s
2 *Pseudomonas spp.*	8 (10.7%)		8 (8.5%)		n.s
	4 (5.3%)	*Pseudomonas aeruginosa*	7 (7.4%)	*Pseudomonas aeruginosa*	n.s
	2 (2.7%)	*Pseudomonas putida*	1 (1.1%)	*Pseudomonas putida*	n.s
	2 (2.7%)	*Pseudomonas mendocina*			n.s
3 *Stenotrophomonas maltophilia*	0		3 (3.2%)		n.s
4 *Acinetobacter baumannii*	0		8 (8.5%)		0.009
5 *Serratia marcescens*	0		3 (3.2%)		n.s
Obligate anaerobe	0		2 (2.1%)		n.s
			1 (1.1%)	*Bacteroides fragilis*	n.s
			1 (1.1%)	*Prevotella*	n.s
*Canidia albicans*	6 (8.0%)		1 (1.1%)		0.045
*Mycobacterium tuberculosis*	1 (1.3%)		0		n.s

*MRSA* Methicillin-resistant S. aureus, *MSSA* Methicillin-sensitive S. aureus, *CoNS* Coagulase-negative Staphylococci, *MRCoNS* Methicillin-resistant CoNS, *MRSE* Methicillin-resistant S. epidermidis, *MRSHo* Methicillin-resistant S. hominis, *MRSH* Methicillin-resistant S. haemolyticus, *MRSC* Methicillin-resistant S. capitis, *MSCoNS* Methicillin-sensitive coagulase-negative Staphylococci, *MSSE* Methicillin-sensitive S. epidermidis, *MSSHo* Methicillin-sensitive S. hominis

In the PJI cohort, the most prevalent pathogens were *Staphylococci* (61.3%), which included coagulase-negative *Staphylococci* (CoNS) and *S. aureus* (32.0% and 29.3%, respectively). Methicillin-resistant CoNS (MRCoNS) accounted for 26.7%, and methicillin-sensitive *S. aureus* (MSSA) accounted for 21.3%, followed by methicillin-resistant *Staphylococcus capitis* (MRSC, 10.7%). GNB were the most frequently identified pathogens in the FRI cohort (56.4%), and *Staphylococci* accounted for 31.9%. The most prevalent GNB was *Enterobacteriaceae* (33.0%), including *Enterobacter spp.* (11.7%) and *Klebsiella pneumoniae* (9.6%).

Fisher’s exact test was used to compare every pathogen distribution between the two cohorts (*p* < 0.001). *Staphylococci* were more common in the PJI cohort than in the FRI cohort (61.3% vs. 31.9%, *p* < 0.001), among which MRCoNS (26.7%, *p* = 0.002) and *Candida albicans* (8.0%, *p* = 0.045) were more frequently detected in the PJI cohort. GNB was more common in the FRI cohort than PJI (56.4% vs. 26.7%, *p* < 0.001). In addition, *Enterobacter cloacae* and *Acinetobacter baumannii* were detected only in the FRI cohort (9.6% and 8.5%, respectively).

### Empirical antimicrobial therapy

**Fig. 2:**
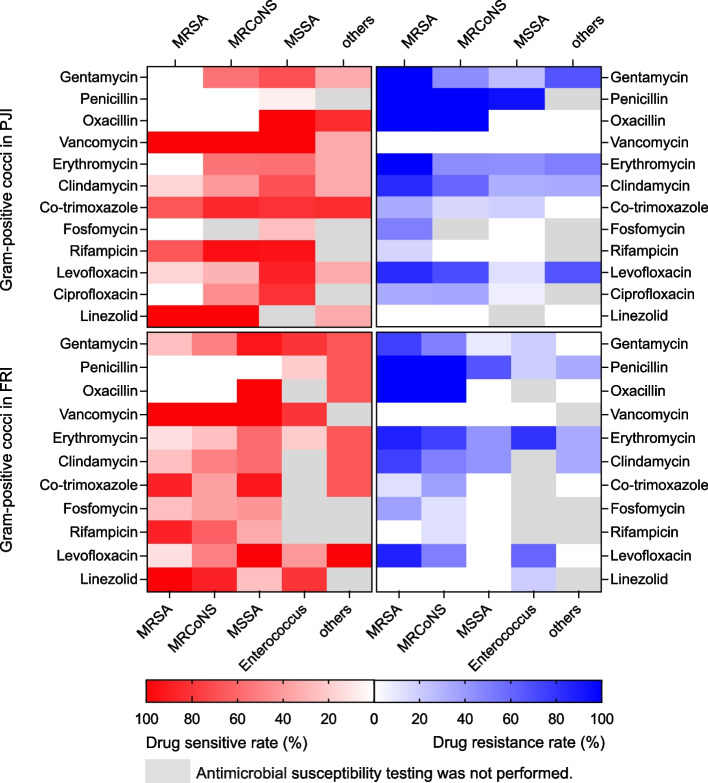
Heatmap showing drug resistance rates and sensitivity rates analysis of Gram-positive cocci in the PJI and FRI cohorts

**Fig. 3:**
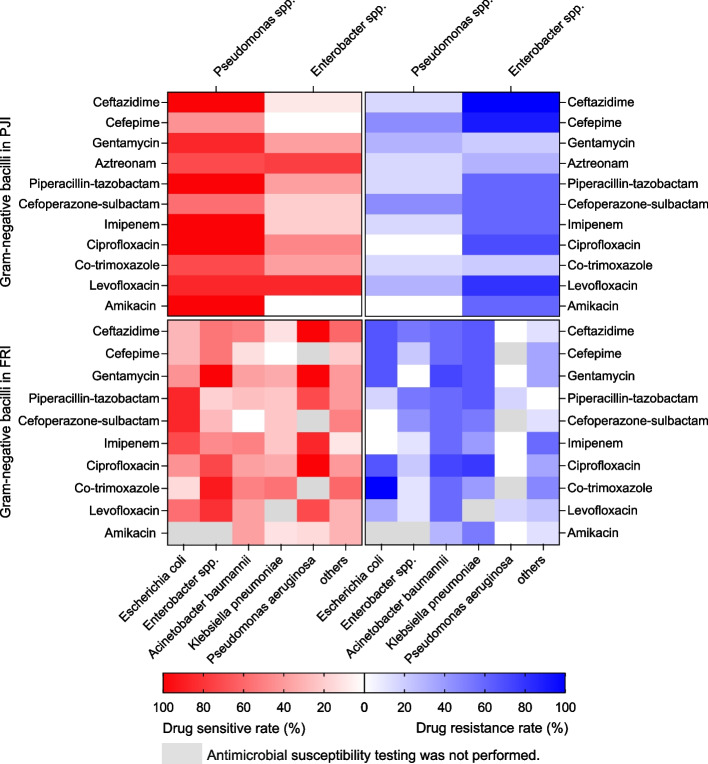
Heatmap showing drug resistance rates and sensitivity rates analysis of Gram-negative bacilli in the PJI and FRI cohorts

We noticed that *Staphylococci* and GNB were responsible for the majority of infections in both the PJI and FRI cohorts. Thus, Pearson’s chi-squared test was carried out and showed significant differences in the distribution of these two types of pathogens between the two cohorts (*p* < 0.001). We conducted predicted analyses against isolated pathogens from PJI and FRI cohorts except fungi and Mycobacterium tuberculosis to evaluate the expected efficacy of three combinations of antibiotics (﻿imipenem + vancomycin, ciprofloxacin + vancomycin, and piperacillin/tazobactam + vancomycin). The combination of these three antibiotics was based on the preliminary analysis of the results of antimicrobial susceptibility testing (Supplemental Tables 3, 4, 5, and 6 and Figs. 2 and 3), combined with the reports confirmed in the previous literature [9–11, 13, 14, 19] and clinical experience obtained from consultation with the Department of Infectious Diseases of our hospital.

**Fig. 4:**
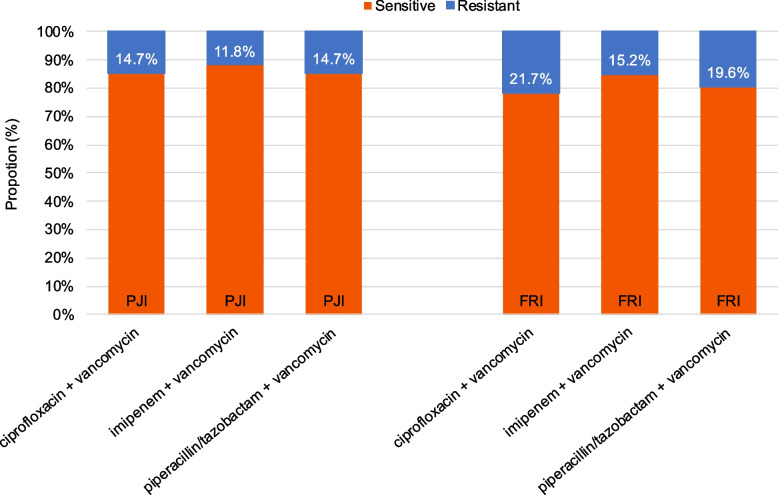
Predicted efficacy of empirical antimicrobial regimens for the PJI and FRI cohorts

The combination of ﻿imipenem + vancomycin showed the broadest antibiotic coverage (88.2%) of isolated pathogens in the PJI cohort, followed by ciprofloxacin + vancomycin and piperacillin/tazobactam + vancomycin, which both achieved 85.3% antibiotic coverage (Fig. 4). In the FRI cohort, ﻿imipenem + vancomycin achieved 84.8% antibiotic coverage of isolated pathogens, followed by piperacillin/tazobactam + vancomycin, which covered 80.4%. A lower sensitivity rate was found for ciprofloxacin + vancomycin (78.3%).

## Discussion

Our study found significant differences in the pathogen distribution of PJI and FRI cohorts, and the most prevalent pathogens in the PJI cohort were *Staphylococci*, while GNB were the most frequently identified pathogens in the FRI cohort. Furthermore, the proportion of these two types of pathogens between the two cohorts showed a significant difference. In addition, MDROs were more frequently detected in the FRI cohort, while MRCoNS and *C. albicans* were more frequently detected in PJI.

### Pathogen distribution

In this study, Gram-positive cocci, especially *Staphylococci*, were the main reason for PJI. A multicenter study analysed the leading pathogens causing PJI in China, and showed that the proportion of Gram-positive cocci was 65.7% [20]. Ares et al. [21] conducted a review of 14 studies showing that *S. aureus* and CoNS both accounted for 27.0% of PJI. Triffault-Fillit et al. [22] disclosed 28.6% CoNS in PJI cases, which is comparable to the 30.4% found in our study. The abovementioned previous literature has reported similar results, which are consistent with our study. In addition, the prevalence of PJI caused by drug-resistant pathogens and fungi is increasing.

Ravi et al. [23] found that the proportion of MRCoNS was 25.6% compared to 26.7% in our PJI cohort. Analysis of PJI microbiological patterns in a large cohort containing 997 patients with PJI and 180 (18.1%) of them were detected with MRCoNS [24]. Charalambous et al. [25] reported a poor rate of success in treating CoNS PJI, which was likely due to the interaction of inherent virulence through biofilm formation and decreased antibiotic efficacy. Regarding fungal infections, *C. albicans* were detected more frequently in the PJI cohort, with a prevalence of 8.0%. *Candida* periprosthetic joint infection (CPJI) is a rare, difficult-to-treat disease. Rarely, PJI may be caused by fungi with a global incidence of 1%, and *Candida* species are responsible for at least 80% of PJI cases [26, 27]. Up to 20% of fungal PJI cases are accompanied by bacterial infections [28]. We found *C. albicans*infection in the PJI cohort, which might be related to the risk factors for fungal infection, including immunosuppression, systemic diseases, and prolonged antimicrobial therapy [29]. *Candida* species often grow as a biofilm adhering to bioprosthetic implants, including cement spacers [30], and this may contribute to the persistence and relapse of the infection [31, 32].

GNB were the most frequent pathogens in our FRI cohort. A multicenter study in Northeast China investigated 744 patients with FRI from 2011 to 2020, and 266 GNB (47.0%) were identified among the detected pathogens. Furthermore, *S. aureus*, *S. epidermidis*, *Pseudomonas aeruginosa*, *Escherichia coli*, *K. pneumoniae*, and *Enterobacter cloacae*were similar to our study of the bacterial species and proportions [33]. Depypere et al. [34] reported that GNB was isolated in 27.8% of FRI cases, which is higher than the 14% generally reported in PJI studies. Nevertheless, some literature from Europe reported that the main pathogens of FRI were *Staphylococci* [12, 34–36]. A higher rate of GNB in our FRI cohort might be due to initial EAT in our institution mainly against Gram-positive bacteria. Another reason might be that the causes of open fractures in China are dominated by traffic accidents and factory mechanical injuries compared with developed countries.

### Multi-drug resistant organisms

In our study, MDROs were detected more frequently in the FRI cohorts. A comparison of bacteria isolated from open fractures following debridement and subsequent infection concluded that 60.8% of postoperative infections were caused by MDROs [37]. Zhang et al. [38] reported that 546 strains of pathogens were detected in FRI patients, with only 105 strains (19.2%) of MDROs. The pathogenesis of FRI was more complex, and patients with FRI were more severely ill, resulting in more bacterial species and higher MDR infection rates in the FRI cohort.

### Specific pathogens in each cohort

*Enterobacter spp.* and *A. baumannii* were only isolated in the FRI cohort. FRI within three weeks after surgery due to *Enterobacter spp.* occurred primarily in lower extremity fractures, especially hip fractures, because patients with these traumas tended to be elderly [39], undertook indwelling urinary catheters, required absolute bed rest, and might couple with inadequate perineal care. These characteristics result in infection caused by colonizing the microbiota of the local skin [40]. Yeramosu et al. [41] enrolled 248 patients who underwent operatively treated pilon fractures and declared that *Enterobacter cloacae* was one of the most common pathogens, accounting for 16.7%.

The prevalence of *A. baumannii* was 12.1%, which was only isolated in the FRI cohort. Hao et al. [37] analysed bacteria isolated from open fractures following debridement and subsequent infection and demonstrated that *A. baumannii* accounted for 49.3%, which indicated that *A. baumannii* was more likely to cause infections in patients with open fractures and severe trauma. Moreover, 87.5% of patients in whom *A. baumannii* was detected underwent severe trauma and therefore had a long duration in the ICU. This might be because *A. baumannii* is an opportunistic human pathogen that predominantly infects critically ill patients [42], so it was entirely concentrated in patients with FRI. In addition, Caricato et al. [43] concluded that the presence of long-term trans-skeletal traction was the only independent risk factor for *A. baumannii* infection (*p* = 0.04). However, we could not isolate *Enterobacter spp.* and *A. baumannii* in the PJI cohort.

### Differences in mechanisms

To determine the reason for the differences in pathogen distribution between the two groups, we compared the mechanisms of these two infections. In the previous literature, PJI occurred through various mechanisms: first, direct seeding from external contaminants or contiguous spread with airborne organisms and those present on the patient’s skin [44]; second, haematogenous spread from other body sites; and third, recurrent infection [45]. FRI generally occurs exogenously due to the trauma itself (e.g., open fractures), during insertion of the fixation device, or disturbed wound healing or late soft tissue coverage in cases of open fractures. Haematogenous infections are rare [46, 47]. Therefore, the differences in mechanisms can partially explain the differences in the pathogen distribution of the two infections. However, the mechanism has not yet been elucidated thoroughly and remains to be further studied.

### Empirical antimicrobial regimens

Previous guidelines in PJI recommend the use of an anti-Gram-positive agent such as vancomycin in combination with broad-spectrum β-lactams such as piperacillin-tazobactam and 3^rd^- or 4.^th^-generation cephalosporins to permit bone tissue penetration [9, 10]. Moran et al. [11] recommended the use of vancomycin combined with a carbapenem as the empirical therapy for PJI. Reported recommendations of empirical antimicrobial regimens used for FRI include a glycopeptide and an agent covering GNB [13]. A recent study recommends that meropenem + vancomycin, gentamicin + vancomycin, and co-amoxiclav + glycopeptide are the best therapeutic options for FRI, regardless of the onset of infection [35].

In our study, *Staphylococci* and GNB were responsible for the majority of infections in both the PJI and FRI cohorts. As a result, we concluded that EAT mainly aimed at these two types of pathogens in PJI and FRI consistently, which is in line with previous literature. The empirical antibiotic regimen for FRI is recommended for piperacillin/tazobactam + vancomycin. For PJI, ciprofloxacin + vancomycin and piperacillin/tazobactam + vancomycin exhibited the same antibiotic coverage, so both can be recommended. However, microbial epidemiology in PJI and FRI depends heavily on the center and the clinical situation [9], making it difficult to provide universal recommendations.

The current empirical antimicrobial regimens cover both Gram-positive cocci and GNB, but they are too broad to be accurate, which tends to result in adverse effects of antibiotic abuse. Although several studies have recommended the use of carbapenems for PJI and FRI, empirical antimicrobial regimens should avoid the containment of reserve antibiotics due to hitherto unknown effects on the development of multi-drug resistance [35]. Once there is evidence of pathogens and their sensitivity, patients should be treated with targeted and de-escalated antimicrobial therapy due to the risk of enhanced antimicrobial resistance by broad-spectrum antimicrobial combination [12, 48]. Local antibiotic carriers (gentamicin + vancomycin) are a feasible approach to avoid the side effects of systemic antimicrobial therapy [12, 49], especially for the treatment of PJI, as antibiotic coverage reached 91.2% in our data. According to previous studies, intraoperative application of implants or cement loaded with the combination of antibiotics is reasonable [50]; however, clinical efficacy needs to be further investigated [51, 52]. Our further research found that the pathogen distribution of *Staphylococci* and GNB showed significant differences between the two cohorts. Therefore, empirical antimicrobial regimens should focus on the coverage of *Staphylococci* in PJI and GNB in FRI, which could guide for the formulation of more accurate empirical antimicrobial regimens.

### Limitations

A retrospective study of only one center and a relatively small cohort may result in a local epidemiological bias. Since the incidence of PJI is relatively low, a long study period is necessary to obtain a large patient cohort. An oft-cited reason for the situation in which antimicrobial susceptibility testing for certain antibiotics can be negative is the administration of antibiotics before treatment in our center and obtaining cultures. In addition, there are inherent differences in the pathogen distribution between implant-associated infections at different sites, mainly because of the specific colonizing bacteria.

## Conclusions

*Staphylococci* and GNB were responsible for the majority of infections in both PJI and FRI, and the distribution of these two types of pathogens showed significant differences between the two cohorts. EAT should focus on the coverage of *Staphylococci* in PJI and GNB in FRI, and infections caused by MDROs should be more vigilant in FRI while the high incidence of MRCoNS in PJI should be noted, which could guide for the formulation of more accurate empirical antimicrobial regimens. Targeted therapy for FRI caused by *A. baumannii* and PJI caused by *C. albicans* needs to be further investigated. At present, there are few studies on the differences in pathogen distribution between PJI and FRI, and mechanistic investigations are rare. Our study reports pathogen differences between these two infections and provides clinical evidence for studies on the mechanism of infection.

## Supplementary Information

**Additional file 1:** **Supplemental table 1.** Comparison of isolated microorganisms in early, delayed and late PJI.

**Additional file 2:** **Supplemental table 2.** Comparison of isolated microorganisms in early, delayed and late FRI.

**Additional file 3:** **Supplemental table 3.** Analysis of drug resistance rates and sensitivity rates of Gram-positive cocci in PJI.

**Additional file 4:** **Supplemental table 4.** Analysis of drug resistance rates and sensitivity rates of Gram-negative bacilli in PJI.

**Additional file 5:** **Supplemental table 5.** Analysis of drug resistance rates and sensitivity rates of Gram-positive cocci in FRI.

**Additional file 6:** **Supplemental table 6.** Analysis of drug resistance rates and sensitivity rates of Gram-negative bacilli in FRI.

**Additional file 7:** **Supplemental Table 7.** Isolated microorganisms in FRI after open fractures and closed fractures.

## Data Availability

All data used and analysed during the current study are available from the corresponding author on reasonable request.

## Abbreviations

FRI: Fracture-related infection
PJI: Periprosthetic joint infection
GNB: Gram-negative bacilli
MDR: Multi-drug resistant
MDRO: Multi-drug resistant organism
EAT: Empirical antimicrobial therapy
THA: Total hip arthroplasty
TKA: Total knee arthroplasty
BMI: Body mass index
CCI: Charlson Comorbidity Index
SD: Standard deviation
MRSA: Methicillin-resistant *Staphylococcus aureus*
MSSA: Methicillin-sensitive *Staphylococcus aureus*
CoNS: Coagulase-negative *Staphylococci*
MRCoNS: Methicillin-resistant CoNS
MRSE: Methicillin-resistant *Staphylococcus epidermidis*
MRSHo: Methicillin-resistant *Staphylococcus hominis*
MRSH: Methicillin-resistant *Staphylococcus haemolyticus*
MRSC: Methicillin-resistant *Staphylococcus capitis*
MSCoNS: Methicillin-sensitive CoNS
MSSE: Methicillin-sensitive *Staphylococcus epidermidis*
MSSHo: Methicillin-sensitive *Staphylococcus hominis*
